# Association between red cell distribution width–and–albumin ratio and the risk of peripheral artery disease in patients with diabetes

**DOI:** 10.3389/fendo.2024.1272573

**Published:** 2024-02-09

**Authors:** Dongling Li, Juan Long, Jialu Zhang, Meinan He, Qingxiang Zeng, Qiaoling He, Wanhua Zhan, Yongqian Chi, Mengchen Zou

**Affiliations:** ^1^ Department of Endocrinology and Metabolism, Nanfang Hospital, Southern Medical University, Guangzhou, China; ^2^ Department of Endocrinology, Central Hospital of Zengcheng District, Guangzhou, China

**Keywords:** red cell distribution width and albumin ratio, peripheral artery disease, diabetes, RDW (red cell distribution width), association

## Abstract

**Aim:**

The aim of this study is to explore the association between red blood cell distribution width–to–albumin ratio (RAR) and the risk of peripheral artery disease (PAD) in patients with diabetes.

**Methods:**

This cross-sectional study extracted the data of 1,125 participants with diabetes from the National Health and Nutrition Examination Survey database. A weighted univariable logistic regression model was used to explore variables associated with PAD. With PAD as the outcome variable, a weighted logistic regression model was established. The odds ratio (OR) and 95% confidence interval (CI) were effect size.

**Results:**

After adjusting for covariates, the risk of PAD in patients with diabetes was observed in those with higher RAR (OR = 1.83; 95% CI: 1.06–3.15). In addition, RAR ≥3.25 was related to increased risk of PAD in patients with diabetes (OR = 2.04; 95% CI: 1.05–3.95). In people with diabetes aged ≥65, RAR was a risk factor for PAD with an OR value of 2.67 (95% CI: 1.30–5.46). RAR ≥3.25 was associated with increased risk of PAD (OR = 3.06; 95% CI: 1.15–8.11) relative to RAR <2.80. In people with diabetes who smoked, the risk of PAD was elevated in those with RAR ≥3.25 (OR = 2.85; 95% CI: 1.28–6.32). As for patients with cardiovascular disease, the risk of PAD was elevated as the increase of RAR (OR = 2.31; 95% CI: 1.05–5.10). RAR ≥3.25 was correlated with increased risk of PAD (OR = 3.75; 95% CI: 1.42–9.87). The area under the curve of RAR for the risk of PAD in patients with diabetes was 0.631 (95% CI: 0.588–0.675).

**Conclusion:**

A higher RAR was related to increased risk of PAD in patients with diabetes. The findings might offer a reference for the management of PAD in patients with diabetes.

## Introduction

Peripheral artery disease (PAD) is a direct macrovascular disorder of diabetes with an estimated prevalence at 20%–28% (1, 2). The risk of developing PAD was increased nearly 30% with each 1% increase in hemoglobin A1c (HbA1c) during the follow-up period (3). Patients with diabetes and PAD had a higher risk of lower limb amputation than patients without diabetes, and patients with PAD had a higher risk of cardiovascular disease (CVD) and mortality (4). In patients with diabetes, PAD develops early and progresses rapidly, but usually has no obvious symptoms (5). Identifying the indicators that are closely related to the risk of PAD in patients with diabetes to help identify those with high risk of PAD in these patients is necessary.

Hemogram parameters were widely reported to be associated with the risk of CVDs (6, 7). Previously, red blood cell distribution width (RDW) was reported to be associated with the severity of chronic kidney disease (CKD), macrovascular and microvascular complications, and all-cause mortality in in patients with diabetes (8, 9). In addition, albumin was also a predictor for the progression of CKD in patients with newly diagnosed type 2 diabetes (10). Recently, a new inflammatory indicator, RDW-to-albumin ratio (RAR), which combines RDW and albumin level, has been used to assess the risk of poor prognosis in some CVDs (11, 12). Another study found that, in patients with diabetes, those with higher RAR were correlated with an increased risk of developing retinopathy (13). At present, whether RAR was associated with the risk of PAD in patients with diabetes was still unclear.

This study aimed to explore the association between the RAR and the risk of PAD in patients with diabetes based on the data from the National Health and Nutrition Examination Survey (NHANES). We conducted subgroup analyses to validate the findings across different patient populations, including those stratified by age, smoking status, and the presence of CVDs.

## Methods

### Study design and population

This cross-sectional study extracted the data of 2,081 patients with diabetes from the NHANES database. Conducted by the Centers for Disease Control and Prevention’s National Center for Health Statistics, the NHANES performed a comprehensive monitoring of the nation’s nutrition and health status through direct physical examinations, clinical and laboratory tests, personal interviews, and related measurement procedures. The examinations are conducted in mobile examination centers that travel to various locations throughout the country, ensuring a standardized environment for the health examinations (14). In our study, diabetes was diagnosed by fasting glucose/HbA1c, physician diagnosis, or those who had anti-diabetic drug. The excluded criteria were 1) <18 years, 2) without measurement of the left or right ankle brachial pressure index (ABPI), and 3) without measurement of RDW or albumin. Finally, 1,125 participants were included.

### Potential confounders and definitions

Age (<65 years or ≥65 years), gender (male or female), race (non-Hispanic White, non-Hispanic Black, or others), education [less than 9th grade, 9th to 11th grade (includes 12th grade with no diploma), high school graduate/general equivalent diploma (GED), or equivalent or some college or Associate of Arts (AA) degree/college graduate or above], poverty-to-income ratio (≤1.0, 1.0–2.0, >2.0, or unknown), marriage (never married, married, or others), physical activity [<450 metabolic equivalent of task (MET) × min/week, ≥450 MET × min/week, or unknown], smoking (yes or no), drinking (<once/week, ≥once/week, or no), hypertension (yes or no), dyslipidemia (yes or no), CVD (yes or no), diabetic retinopathy (yes or no), CKD (yes or no), family history of CVD (yes or no), body mass index (BMI) (<25 kg/m^2^, 25 kg/m^2^ to 30 kg/m^2^, or ≥30 kg/m^2^), waist circumference (cm), energy (kcal), hemoglobin (g/dL), C-reaction protein (mg/dL), anti-platelet drug (yes or no), anti-coagulants drug (yes or no), adrenal cortical steroids (yes or no), and diabetes drug (yes or no).

Physical activity was converted into energy consumption based on the questionnaire in the database. Energy consumption (MET × min) = recommended MET × exercise time of corresponding activity (min), which can be converted into weekly energy consumption, and divided into three categories including <450 MET × min/week, ≥450 MET × min/week, and unknown. BMI was grouped into normal (18.5 kg/m^2^ to 24.9 kg/m^2^), underweight (<8.5 kg/m^2^), overweight (25 kg/m^2^ to 29.9 kg/m^2^), and obese (≥30 kg/m^2^). Because there were only two people in the underweight group, the underweight group was combined with the normal group.

### Main and outcome variables

The RAR was the main variable that was calculated on the basis of RAR. The continuous variable and the categorical variable of RAR were used for analysis. As a categorical variable, RAR was divided into <2.80, 2.80–2.98, 2.98–3.25, and ≥3.25 according to quarters. RDW was divided into <12.18%, 12.18%–12.56%, 12.56%–13.09%, and ≥13.09%, whereas albumin was divided into <3.95 g/dL, 3.95 g/dL to 4.19 g/dL, 4.19 g/dL to 4.39 g/dL, and ≥4.39 g/dL according to the respective quarters. PAD was the outcome, which was diagnosed on the basis of the left or right ABPI <0.9 (15).

### Statistical analysis

Kolmogorov–Smirnov normality test was used for quantitative data. Normally distributed measurement data were described as mean (standard error) [mean (SE)], independent sample t-test was used for comparison between two groups, and analysis of variance was used for comparison between multiple groups. Non-normally distributed measurement data were described as median and quartiles [M (Q_1_, Q_3_)], and Kruskal–Wallis test was used for comparison among groups. The enumeration data were described as the numbers and percentages of cases [n (%)], Chi-square test was used for comparison between groups, and rank sum test was used for rank data. A weighted univariable logistic regression model was used to explore variables associated with PAD. With PAD as the outcome variable, a weighted logistic backward regression model was established. In Model I, no variable was adjusted; in Model II, age and gender were adjusted; and in Model III, age, gender, education, poverty-to-income ratio, smoking status, CVD, CKD, and anti-tuberculosis drug were adjusted. Missing values were manipulated via random forest using python miceforest package for interpolation processing (**Supplementary Table 1**). Poverty-to-income ratio and physical activity had missing values >5%, and the missing values were classified as the unknown group. Sensitivity analysis was performed to compare the missing data before and after interpolation (**Supplementary Table 2**). Subgroup analysis was stratified on the basis of age, CVD, and smoking status. The odds ratio (OR) and 95% confidence interval (CI) were effect size. Receiver operator characteristic curves of RAR, RDW, and albumin for the risk of PAD in patients with diabetes were plotted, and the areas under the curve (AUCs) were calculated and compared via Delong test. All statistical tests were conducted by a two-sided test with the test level α = 0.05. Python 3.9 was used for missing value processing, and SAS 9.4 (SAS Institute Inc., Cary, NC, USA) was used for model statistical analysis.

## Results

### Comparisons of the characteristics between the PAD group and the non-PAD group

**Figure 1:**
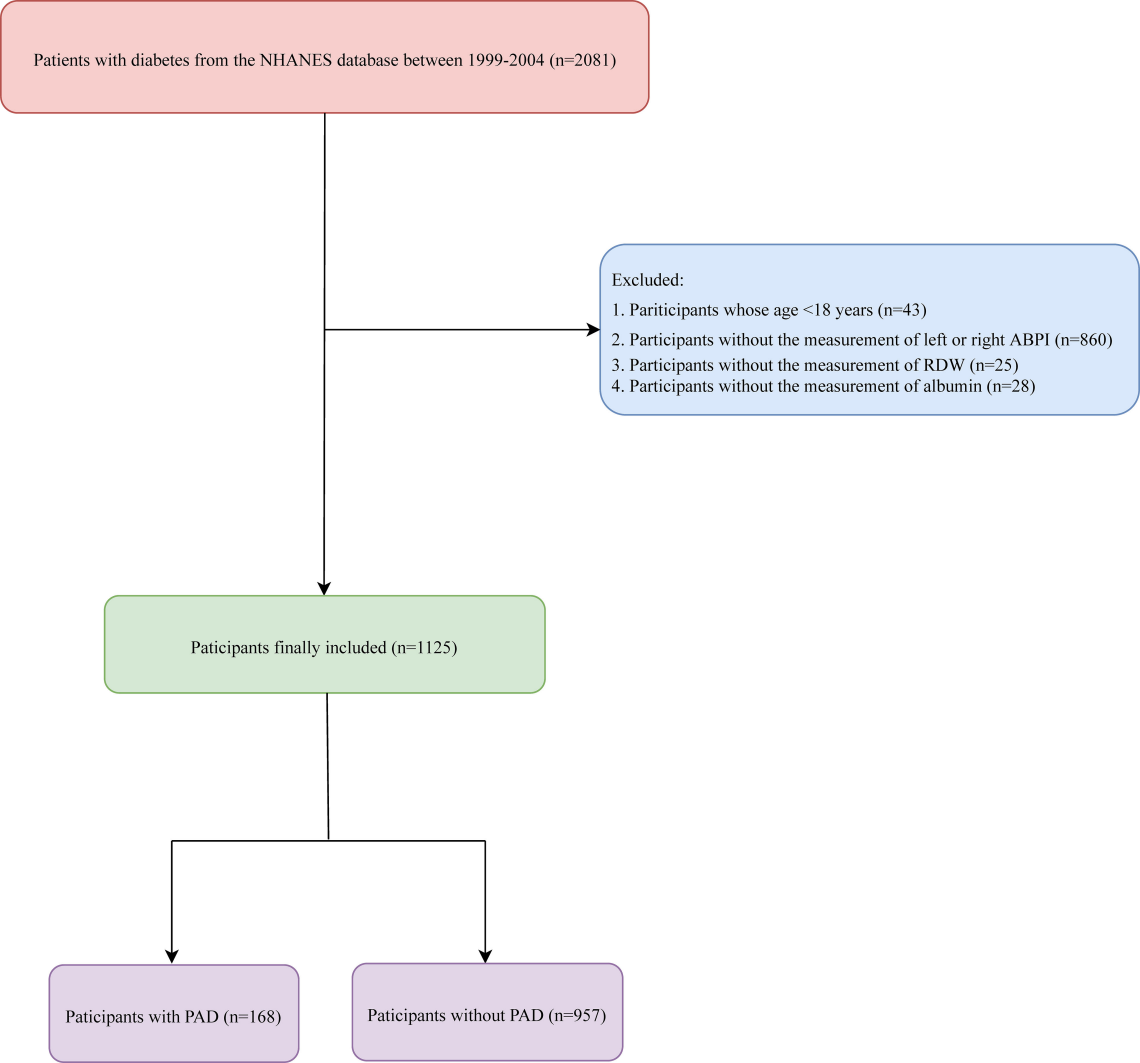
The screen process of the participants.

In total, the data of 2,081 patients with diabetes were retrieved from the NHANES database. Among them, participants whose age <18 years (n = 43), patients without measurement of left or right ABPI (n = 860), and those without measurement of RDW (n = 25) or albumin (n = 28) were excluded. Finally, 1,125 participants were included. The screen process is displayed in **Figure 1**.

The mean RAR in the PAD group was higher than that in the non-PAD group (3.32 vs. 3.04). The percentages of patients with different RAR levels in the PAD group were different compared with that in the non-PAD group. The mean RDW in the PAD group was higher than that in the non-PAD group (13.50% vs. 12.78%). The mean albumin in the PAD group was lower than that in the non-PAD group (4.11 g/dL vs. 4.23 g/dL). The percentages of subjects with CKD in the PAD group was higher than that in the non-PAD group (27.88% vs. 5.07%). More detailed information is presented in **Table 1**.

**Table 1:** Comparisons of the characteristics between the PAD group and the non-PAD group.

Variables	Total (n = 1,125)	Non-PAD (n = 957)	PAD (n = 168)	Statistics	*P*
RAR, mean (SE)	3.08 (0.01)	3.04 (0.01)	3.32 (0.06)	t = −4.50	<0.001
RAR groups, n (%)				χ^2 =^ 27.97	<0.001
<2.80	246 (24.86)	226 (26.19)	20 (14.85)		
2.80–2.98	259 (24.34)	231 (25.34)	28 (16.80)		
2.98–3.25	282 (25.67)	243 (26.19)	39 (21.78)		
≥3.25	338 (25.12)	257 (22.29)	81 (46.56)		
RDW, %, mean (SE)	12.87 (0.04)	12.78 (0.03)	13.50 (0.17)	t = −4.17	<0.001
Albumin, g/dL, mean (SE)	4.22 (0.01)	4.23 (0.01)	4.11 (0.03)	t = 3.52	0.001
Age, years, mean (SE)	60.23 (0.43)	59.05 (0.42)	69.14 (1.12)	t = −9.65	<0.001
Age, n (%)				χ^2 =^ 37.33	<0.001
<65	591 (62.45)	551 (66.81)	40 (29.48)		
≥65	534 (37.55)	406 (33.19)	128 (70.52)		
Gender, n (%)				χ^2 =^ 0.03	0.870
Male	642 (56.89)	542 (56.98)	100 (56.21)		
Female	483 (43.11)	415 (43.02)	68 (43.79)		
Race, n (%)				χ^2 =^ 20.30	<0.001
Non-Hispanic White	462 (66.88)	385 (66.07)	77 (73.05)		
Non-Hispanic Black	248 (12.54)	194 (11.67)	54 (19.15)		
Others	415 (20.57)	378 (22.27)	37 (7.80)		
Education, n (%)				χ^2 =^ 17.07	<0.001
Less than 9th grade	282 (12.34)	225 (10.70)	57 (24.72)		
9th–11th grade (includes 12th grade with no diploma)	221 (17.87)	185 (17.81)	36 (18.32)		
High school graduate/GED or equivalent	241 (24.99)	205 (24.76)	36 (26.72)		
Some college or AA degree/College graduate or above	381 (44.81)	342 (46.74)	39 (30.23)		
Poverty-to-income ratio, n (%)				χ^2 =^ 19.85	<0.001
≤1.0	209 (12.83)	166 (12.07)	43 (18.54)		
1.0–2.0	326 (23.89)	266 (21.88)	60 (39.10)		
>2.0	488 (53.59)	435 (55.85)	53 (36.53)		
Unknown	102 (9.69)	90 (10.20)	12 (5.82)		
Marriage, n (%)				χ^2 =^ 5.37	0.068
Never married	61 (6.78)	56 (7.08)	5 (4.54)		
Married	692 (64.60)	600 (65.87)	92 (55.02)		
Others	372 (28.61)	301 (27.05)	71 (40.44)		
Physical activity, n (%)				χ^2 =^ 15.21	<0.001
<450 MET × min/week	235 (25.79)	214 (26.98)	21 (16.81)		
≥450 MET × min/week	253 (24.80)	223 (25.95)	30 (16.10)		
Unknown	637 (49.42)	520 (47.07)	117 (67.10)		
Smoking, n (%)				χ^2 =^ 13.48	<0.001
No	481 (44.12)	435 (46.64)	46 (25.13)		
Yes	644 (55.88)	522 (53.36)	122 (74.87)		
Drinking, n (%)				χ^2 =^ 5.14	0.077
No	424 (37.22)	365 (37.91)	59 (32.01)		
<Once/week	518 (44.43)	432 (42.93)	86 (55.74)		
≥Once/week	183 (18.35)	160 (19.16)	23 (12.25)		
Hypertension, n (%)				χ^2 =^ 17.41	<0.001
No	253 (26.32)	240 (28.80)	13 (7.57)		
Yes	872 (73.68)	717 (71.20)	155 (92.43)		
Dyslipidemia, n (%)				χ^2 =^ 1.00	0.317
No	118 (9.43)	104 (9.76)	14 (6.98)		
Yes	1007 (90.57)	853 (90.24)	154 (93.02)		
CVD, n (%)				χ^2 =^ 23.38	<0.001
No	654 (59.93)	585 (63.54)	69 (32.68)		
Yes	471 (40.07)	372 (36.46)	99 (67.32)		
Diabetic retinopathy, n (%)				χ^2 =^ 0.31	0.576
No	943 (85.26)	801 (85.51)	142 (83.40)		
Yes	182 (14.74)	156 (14.49)	26 (16.60)		
CKD, n (%)				χ^2 =^ 56.06	<0.001
No	1024 (92.26)	898 (94.93)	126 (72.12)		
Yes	101 (7.74)	59 (5.07)	42 (27.88)		
Family history of CVD, n (%)				χ^2 =^ 0.17	0.685
No	991 (83.68)	840 (83.44)	151 (85.47)		
Yes	134 (16.32)	117 (16.56)	17 (14.53)		
Body mass index, n(%)				χ^2 =^ 4.25	0.119
<25 kg/m^2^	176 (15.13)	143 (15.15)	33 (15.04)		
25 kg/m^2^ to 30kg/m^2^	425 (33.74)	350 (32.59)	75 (42.45)		
≥30 kg/m^2^	524 (51.12)	464 (52.26)	60 (42.51)		
Waist circumference, cm, mean (SE)	107.59 (0.76)	107.64 (0.82)	107.23 (1.29)	t = 0.30	0.769
Energy, kcal, mean (SE)	1963.35 (38.14)	1993.42 (39.82)	1736.32 (126.16)	t = 2.03	0.048
Hemoglobin, g/dL, mean (SE)	14.52 (0.08)	14.60 (0.08)	13.94 (0.18)	t = 3.92	<0.001
C-reaction protein, mg/dL, mean (SE)	0.61 (0.03)	0.57 (0.03)	0.89 (0.21)	t = −1.48	0.145
Anti-platelet drug, n (%)				χ^2 =^ 23.71	<0.001
No	1061 (94.92)	916 (96.54)	145 (82.66)		
Yes	64 (5.08)	41 (3.46)	23 (17.34)		
Anti-coagulants drug, n (%)				χ^2 =^ 7.38	0.007
No	1088 (96.81)	932 (97.49)	156 (91.66)		
Yes	37 (3.19)	25 (2.51)	12 (8.34)		
Adrenal cortical steroids, n (%)				χ^2 =^ 0.02	0.888
No	1092 (97.08)	928 (97.05)	164 (97.31)		
Yes	33 (2.92)	29 (2.95)	4 (2.69)		
Anti-tuberculosis drug, n (%)					
No	1124 (99.98)	956 (99.98)	168 (100.00)		
Yes	1 (0.02)	1 (0.02)	0 (0.00)		
Diabetes drug, n (%)				χ^2 =^ 5.24	0.022
No	396 (38.21)	354 (39.75)	42 (26.60)		
Yes	729 (61.79)	603 (60.25)	126 (73.40)		

PAD, peripheral arterial diseases; SE, standard error; RAR, the red blood cell distribution width–to–albumin ratio; RDW, red blood cell distribution width; CVD, cardiovascular disease; CKD, chronic kidney disease; GED, general equivalent diploma; AA, Associate of Arts.

### Association between the RAR and the risk of PAD in patients with diabetes

**Figure 2:**
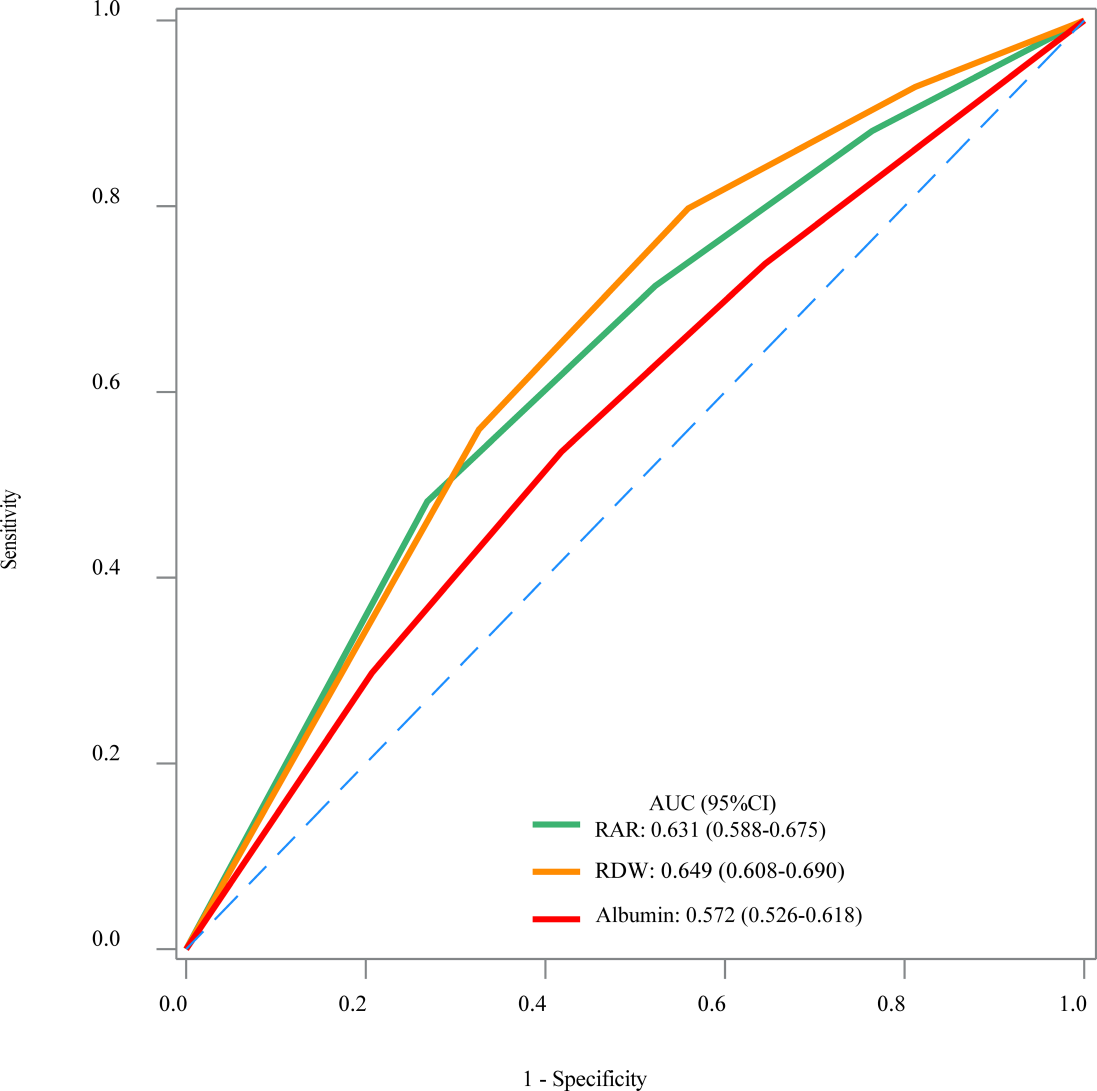
The receiver operator characteristic curves of the RAR, RDW, and albumin for the risk of PAD in patients with diabetes.

The results of weighted univariable logistic regression model revealed that age (OR = 4.82; 95% CI: 2.91–7.97), race (OR = 0.32; 95% CI: 0.17–0.60), education, poverty-to-income ratio, physical activity (OR = 2.29; 95% CI: 1.38–3.79), smoking status (OR = 2.60; 95% CI: 1.54–4.41), hypertension (OR = 4.94; 95% CI: 2.20–11.07), CVD (OR = 3.59; 95% CI: 2.08–6.21), CKD (OR = 7.23, 95% CI: 4.00–13.09), hemoglobin (OR = 0.76; 95% CI: 0.66–0.88), C-reaction protein (OR = 1.17; 95% CI: 1.04–1.32), anti-platelet drug (OR = 5.85; 95% CI: 2.60–13.15), anti-coagulants drug (OR = 3.54; 95% CI: 1.28–9.79), and diabetes drug (OR = 1.82; 95% CI: 1.06–3.13) were potential covariates associated with the risk of PAD in patients with diabetes (**Supplementary Table 3**). Backward stepwise regression data indicated that age, gender, education, poverty-to-income ratio, smoking status, CVD, CKD, and anti-tuberculosis drug were covariates. In the crude model, increased RAR might be associated with the elevated risk of PAD in patients with diabetes. Compared with the RAR <2.80 group, RAR ≥3.25 might increase the risk of PAD in patients with diabetes. After adjusting for covariates, the risk of PAD in patients with diabetes was observed in those with higher RAR (OR = 1.83; 95% CI: 1.06–3.15). In addition, RAR ≥3.25 was related to the increased risk of PAD in patients with diabetes (OR = 2.04; 95% CI: 1.05–3.95) (**Table 2**). No significant association between RDW and the risk of PAD in patients with diabetes was observed (*P* > 0.005). Moreover, the association between albumin and the risk of PAD in patients with diabetes was not statistically different (*P* > 0.005) (**Table 2**). The AUC of RAR (AUC = 0.631; 95% CI: 0.588–0.675) for the risk of PAD in patients with diabetes was higher than that of albumin (AUC = 0.572; 95% CI: 0.526–0.618) (**Figure 2**, **Table 3**). No significant difference was found between the AUC of RAR and that of RDW (AUC = 0.649; 95% CI: 0.608–0.690) for the risk of PAD in patients with diabetes (*P* > 0.05) (**Figure 2**, **Table 3**).

**Table 2:** Association between the RAR and the risk of PAD in patients with diabetes.

Variables	Model I		Model II		Model III	
	OR (95% CI)	*P*	OR (95% CI)	*P*	OR (95% CI)	*P*
RAR	3.24 (1.94–5.41)	<0.001	2.92 (1.84–4.64)	<0.001	1.83 (1.06–3.15)	0.030
RAR groups						
<2.80	Ref		Ref		Ref	
2.80–2.98	1.17 (0.51–2.70)	0.708	1.06 (0.47–2.42)	0.880	1.03 (0.45–2.35)	0.942
2.98–3.25	1.47 (0.71–3.03)	0.293	1.24 (0.59–2.63)	0.565	0.93 (0.45–1.94)	0.848
≥3.25	3.68 (1.87–7.25)	<0.001	3.06 (1.52–6.14)	0.002	2.04 (1.05–3.95)	0.036
RDW	1.52 (1.22–1.91)	<0.001	1.43 (1.18–1.74)	<0.001	1.22 (0.99–1.50)	0.059
RDW						
<12.18	Ref		Ref		Ref	
12.18–12.56	1.43 (0.48–4.31)	0.514	1.18 (0.36–3.85)	0.777	1.00 (0.31–3.22)	0.995
12.56–13.09	2.03 (0.77–5.30)	0.146	1.61 (0.60–4.35)	0.340	1.42 (0.53–3.80)	0.475
≥13.09	4.80 (2.03–11.35)	<0.001	3.63 (1.48–8.86)	0.006	2.19 (0.91–5.28)	0.079
Albumin	0.34 (0.19–0.61)	<0.001	0.34 (0.18–0.65)	0.002	0.55 (0.30–1.01)	0.053
Albumin						
<3.95	Ref		Ref		Ref	
3.95–4.19	0.87 (0.43–1.73)	0.677	0.83 (0.41–1.65)	0.578	1.01 (0.46–2.21)	0.987
4.19–4.39	0.41 (0.22–0.76)	0.006	0.44 (0.23–0.84)	0.013	0.62 (0.32–1.19)	0.146
≥4.39	0.47 (0.29–0.77)	0.004	0.48 (0.27–0.84)	0.012	0.65 (0.36–1.19)	0.161

PAD, peripheral arterial diseases; RAR, the red blood cell distribution width–to–albumin ratio; RDW, red blood cell distribution width; OR, odds ratio; CI, confidence interval; Ref, reference; CVD, cardiovascular disease; CKD, chronic kidney disease.

Model I: Weighted univariable logistic regression model.

Model II: Weighted multivariable logistic regression model adjusted for age and gender.

Model III: Weighted multivariable logistic regression model adjusted for age, gender, education, poverty-to-income ratio, smoking status, CVD, CKD, and anti-tuberculosis drug.

**Table 3:** The AUC of the RAR for the risk of PAD in patients with diabetes.

Variables	AUC (95% CI)	Delong test
RAR	0.631 (0.588–0.675)	Ref
RDW	0.649 (0.608–0.690)	0.2937
Albumin	0.572 (0.526–0.618)	<0.001

PAD, peripheral arterial diseases; RAR, the red blood cell distribution width–to–albumin ratio; RDW, red blood cell distribution width; AUC, area under the curve; CI, confidence interval; Ref, reference.

### Subgroup analysis of the association between the RAR and the risk of PAD in patients with diabetes

In people with diabetes aged ≥65, RAR was a risk factor for PAD with an OR value of 2.67 (95% CI: 1.30–5.46). RAR ≥3.25 was associated with the increased risk of PAD (OR = 3.06; 95% CI: 1.15–8.11) relative to RAR <2.80. No significant association between the RAR and the risk of PAD was found in patients with diabetes <65 years (*P* > 0.05). In people with diabetes who smoked, the risk of PAD was elevated in those with RAR ≥3.25 (OR = 2.85; 95% CI: 1.28–6.32). The association between the RAR and the risk of PAD was not statistically different in non-smoking patients with diabetes (*P* > 0.05). As for patients with CVD, the risk of PAD was elevated as the increase of RAR (OR = 2.31; 95% CI: 1.05–5.10). RAR ≥3.25 was correlated with the increased risk of PAD (OR = 3.75; 95% CI: 1.42–9.87). No association was identified in people with diabetes who are not complicated with CVD (*P* > 0.05) (**Table 4**).

**Table 4:** Subgroup analysis of the association between the RAR and the risk of PAD in patients with diabetes.

Variables	n	OR (95% CI)	*P*	n	OR (95% CI)	*P*
Subgroup I: Age		Age < 65 (n = 591)			Age ≥ 65 (n = 534)	
RAR		1.09 (0.47–2.52)	0.840		2.67 (1.30–5.46)	0.008
RAR groups						
<2.80	158	Ref		88	Ref	
2.80–2.98	147	0.86 (0.19–3.88)	0.845	112	1.41 (0.53–3.78)	0.486
2.98–3.25	131	0.59 (0.16–2.14)	0.416	151	1.50 (0.57–3.91)	0.402
≥3.25	155	1.64 (0.55–4.85)	0.366	183	3.06 (1.15–8.11)	0.026
Subgroup II: Smoking status		No (n = 481)			Yes (n = 644)	
RAR		1.16 (0.56–2.42)	0.680		2.14 (0.93–4.97)	0.074
RAR groups						
<2.80	100	Ref		146	Ref	
2.80–2.98	115	0.44 (0.09–2.08)	0.293	144	1.40 (0.50–3.96)	0.515
2.98–3.25	118	1.21 (0.28–5.18)	0.794	164	0.91 (0.40–2.08)	0.816
≥3.25	148	1.06 (0.35–3.21)	0.917	190	2.85 (1.28–6.32)	0.011
Subgroup III: CVD		No (n = 654)			Yes (n = 471)	
RAR		1.10 (0.42–2.88)	0.847		2.31 (1.05–5.10)	0.038
RAR groups						
<2.80	172	Ref		74	Ref	
2.80–2.98	171	0.71 (0.23–2.16)	0.539	88	1.50 (0.59–3.83)	0.388
2.98–3.25	161	0.74 (0.26–2.08)	0.556	121	1.24 (0.45–3.44)	0.675
≥3.25	150	0.84 (0.23–3.01)	0.779	188	3.75 (1.42–9.87)	0.009

PAD, peripheral arterial diseases; RAR, the red blood cell distribution width–to–albumin ratio; OR, odds ratio; CI, confidence interval; Ref, reference; CVD, cardiovascular disease; CKD, chronic kidney disease.

Subgroup I: Weighted multivariable logistic regression model adjusted for gender, education, poverty-to-income ratio, smoking status, CVD, CKD, and anti-tuberculosis drug.

Subgroup II: Weighted multivariable logistic regression model adjusted for age, gender, education, poverty-to-income ratio, CVD, CKD, and anti-tuberculosis drug.

Subgroup III: Weighted multivariable logistic regression model adjusted for age, gender, education, poverty-to-income ratio, smoking status, CKD, and anti-tuberculosis drug.

## Discussion

This study evaluated the association between the RAR and the risk of PAD in patients with diabetes. The results delineated that the increased RAR was correlated with the higher risk of PAD in patients with diabetes. RAR ≥3.25 was related to the increased risk of PAD in patients with diabetes compared with that in the RAR <2.80 group. Subgroup analysis revealed that RAR ≥3.25 was associated with the increased risk of PAD in people with diabetes aged ≥65 years, who smoked, and who are complicated with CVD. The findings might provide a reference for the better management of PAD in diabetes patients.

RDW was a routinely available inflammatory marker that was reported to be an independent prognostic marker in patients with PAD (16). Elevated RDW was found to be a predictor of cardiovascular outcomes in extensive aortoiliac disease (17). Sincer et al. found that patients with inadequate coronary collateral development had significantly higher RDW levels compared to patients with adequate coronary collateral development, and RDW was significantly associated with Rentrop collateral grading (18). Zalawadiya et al. indicated that the higher levels of RDW were independently associated with a higher risk of PAD and had a better predictive value for PAD than those in the American College of Cardiology/American Heart Association–defined PAD screening criteria (19). Albumin was also delineated to be associated with PAD in some studies. The increased adjusted ischemia–modified albumin levels were identified as predictors of the presence and severity of PAD (20). Ding et al. elucidated that serum albumin was associated with the risk of PAD in patients with hypersensitivity (21). As a new combined parameter, RAR was previously found to be associated with diabetes-related complications such as diabetic nephropathy and microvascular complications (22, 23). RAR was also identified to be correlated with all-cause mortality in patients with type 2 diabetes and foot ulcers (24). In the present study, RAR was found to be associated with the risk of PAD in patients with diabetes. The increased risk of PAD was observed in diabetes patients with RAR ≥3.25.

The possible mechanisms for the association of the RAR and the risk of PAD in patients with diabetes might due to the inflammatory response (25–27). Inflammation contributes to higher RDW, promoting red cell apoptosis and erythropoietin resistance and reducing erythropoietin production and bioavailability of iron (28). Oxidative stress induces increased RDW by shortening the life span of erythrocytes and increasing the migration of premature erythrocytes to the peripheral circulation (29). Serum albumin exerts anti‐inflammatory and antioxidant properties, and lower serum albumin was associated with increased risk of inflammation, the main mechanism of impaired vascular function (30). Subgroup analysis showed that RAR ≥3.25 was associated with the increased risk of PAD in people with diabetes aged ≥65 years, who smoked, and who are complicated with CVD. Age was widely accepted to be a risk factor for PAD, and people with older age were associated with the higher risk of PAD (31). Smoking status was reported to be associated with low serum albumin levels, as reported in the previous studies, and was also a risk factor for PAD, which increased oxidative stress and inflammation and induced endothelial dysfunction (32, 33). RAR seems to have the potential to provide a risk stratification in patients with diabetes.

This study evaluated the association between the RAR and the risk of PAD in patients with diabetes using multi-stage complex sampling, and the sample representativeness was good. RDW and albumin are routinely measured as part of the extensively used complete blood counts, and they would not require any additional cost, providing a simple and feasible tool for PAD risk identification in patients with diabetes. Some limitations existed in our study. Firstly, the history of diseases and other data were obtained through questionnaires, which might have recall bias. Secondly, because of the limitation of the NHANES, more detailed treatment information and other possible confounding factors were not included. Thirdly, the measurement of the left or right ABPI used for PAD diagnosis was only performed during 1999–2004; thus, the sample size of PAD was small. Further well‐designed prospective cohort studies with adequate sample size are needed to determine the causal association and to clarify the potential underlying mechanisms of the RAR and the risk of PAD in patients with diabetes.

## Conclusions

The current study explores the association between the RAR and the risk of PAD in patients with diabetes and found that a higher RAR was related to the increased risk of PAD in patients with diabetes. The findings might offer a reference for the management of PAD in patients with diabetes.

## Data availability statement

The original contributions presented in the study are included in the article/**Supplementary Material**. Further inquiries can be directed to the corresponding author.

## Ethics statement

The requirement of ethical approval was waived by Nanfang Hospital, Southern Medical University for the studies involving humans because the data was accessed from a publicly available database. The studies were conducted in accordance with the local legislation and institutional requirements. The ethics committee/institutional review board also waived the requirement of written informed consent for participation from the participants or the participants’ legal guardians/next of kin because retrospective nature of the study.

## Author contributions

DL: Conceptualization, Supervision, Writing – original draft, Writing – review & editing. JL: Data curation, Formal Analysis, Methodology, Writing – review & editing. JZ: Data curation, Formal Analysis, Methodology, Writing – review & editing. MH: Data curation, Formal Analysis, Methodology, Writing – review & editing. QZ: Data curation, Formal Analysis, Methodology, Writing – review & editing. QH: Data curation, Formal Analysis, Methodology, Writing – review & editing. WZ: Data curation, Formal Analysis, Methodology, Writing – review & editing. YC: Data curation, Formal Analysis, Methodology, Writing – review & editing. MZ: Conceptualization, Funding acquisition, Project administration, Writing – review & editing.
