# Supplementary material for: Association between red cell distribution width–and–albumin ratio and the risk of peripheral artery disease in patients with diabetes
Source: Front Endocrinol (Lausanne). 2024 Feb 9;15:1272573. doi: 10.3389/fendo.2024.1272573 (PMC10884210; doi:10.3389/fendo.2024.1272573)
Supplement: Supplementary file 1 [file Table_1.docx]

Supplementary Table 1 The percentages of missing values

| Variables | n (%) |
| --- | --- |
| Poverty-to-income ratio | 102 (9.07%) |
| Marriage | 34 (3.02%) |
| Physical activity | 637 (56.62%) |
| Drink | 28 (2.49%) |
| Family history of CVD | 48 (4.27%) |
| Body mass index | 19 (1.69%) |
| Waist circumference | 22 (1.96%) |
| Energy | 32 (2.84%) |

CVD: cardiovascular disease

Supplementary Table 2 Sensitivity analysis was performed to compare the missing data before and after interpolation

| Variables | Before interpolation | After interpolation | Statistics | P |
| --- | --- | --- | --- | --- |
| Marriage, n(%) |  |  | χ^2^=3.36 | 0.186 |
| Never married | 61 (7.02) | 61 (6.78) |  |  |
| Married | 669 (64.33) | 692 (64.60) |  |  |
| Others | 361 (28.64) | 372 (28.61) |  |  |
| Drink, n(%) |  |  | χ^2^=5.70 | 0.058 |
| No | 411 (37.12) | 424 (37.22) |  |  |
| <once/week | 503 (44.09) | 518 (44.43) |  |  |
| ≥ once/ week | 183 (18.78) | 183 (18.35) |  |  |
| Family history of CVD, n(%) |  |  | χ^2^=0.19 | 0.664 |
| No | 948 (83.89) | 991 (83.68) |  |  |
| Yes | 129 (16.11) | 134 (16.32) |  |  |
| Body mass index, kg/m^2^, Mean (S.E) | 31.25 (0.31) | 31.25 (0.30) | t=-0.07 | 0.945 |
| Waist circumference, cm, Mean (S.E) | 107.44 (0.78) | 107.59 (0.76) | t=-1.18 | 0.245 |
| Energy, kcal, Mean (S.E) | 1960.61 (38.27) | 1963.35 (38.14) | t=-0.59 | 0.561 |

S.E: standard error; CVD: cardiovascular disease

Supplementary Table 3 Weighted univariable logistic regression model screening the potential cofounders

| Variables | OR (95% CI) | *P* |
| --- | --- | --- |
| Age |  |  |
| <65 | Ref |  |
| ≥65 | 4.82 (2.91-7.97) | <0.001 |
| Gender |  |  |
| Male | Ref |  |
| Female | 1.03 (0.70-1.52) | 0.870 |
| Race |  |  |
| Non-Hispanic White | Ref |  |
| Non-Hispanic Black | 1.48 (0.92-2.39) | 0.103 |
| Others | 0.32 (0.17-0.60) | <0.001 |
| Education |  |  |
| Less than 9th grade | Ref |  |
| 9-11th grade (Includes 12th grade with no diploma) | 0.45 (0.25-0.79) | 0.007 |
| High school graduate/GED or equivalent | 0.47 (0.25-0.87) | 0.017 |
| Some college or AA degree/College graduate or above | 0.28 (0.15-0.53) | <0.001 |
| Poverty-to-income ratio |  |  |
| ≤1.0 | Ref |  |
| 1.0-2.0 | 1.16 (0.62-2.17) | 0.627 |
| >2.0 | 0.43 (0.22-0.82) | 0.012 |
| Unknown | 0.37 (0.15-0.90) | 0.030 |
| Marriage |  |  |
| Never married | Ref |  |
| Married | 1.30 (0.31-5.51) | 0.714 |
| Others | 2.33 (0.56-9.69) | 0.238 |
| Physical activity |  |  |
| <450 met×minutes/week | Ref |  |
| ≥450 met×minutes/week | 1.00 (0.52-1.91) | 0.990 |
| Unknown | 2.29 (1.38-3.79) | 0.002 |
| Smoke |  |  |
| No | Ref |  |
| Yes | 2.60 (1.54-4.41) | <0.001 |
| Drink |  |  |
| No | Ref |  |
| <once/week | 1.54 (0.87-2.71) | 0.132 |
| ≥ once/ week | 0.76 (0.36-1.61) | 0.461 |
| Hypertension |  |  |
| No | Ref |  |
| Yes | 4.94 (2.20-11.07) | <0.001 |
| Dyslipidemia |  |  |
| No | Ref |  |
| Yes | 1.44 (0.68-3.05) | 0.334 |
| CVD |  |  |
| No | Ref |  |
| Yes | 3.59 (2.08-6.21) | <0.001 |
| Diabetic retinopathy |  |  |
| No | Ref |  |
| Yes | 1.17 (0.66-2.10) | 0.578 |
| CKD |  |  |
| No | Ref |  |
| Yes | 7.23 (4.00-13.09) | <0.001 |
| Family history of CVD |  |  |
| No | Ref |  |
| Yes | 0.86 (0.40-1.85) | 0.688 |
| Body mass index |  |  |
| <25kg/m^2^ | Ref |  |
| 25 kg/m^2-^30kg/m^2^ | 1.31 (0.73-2.35) | 0.351 |
| ≥30kg/m^2^ | 0.82 (0.49-1.36) | 0.432 |
| Waist circumference | 1.00 (0.99-1.01) | 0.769 |
| Energy | 1.00 (1.00-1.00) | 0.106 |
| Hemoglobin | 0.76 (0.66-0.88) | <0.001 |
| C-reaction protein | 1.17 (1.04-1.32) | 0.013 |
| Anti-platelet drug |  |  |
| No | Ref |  |
| Yes | 5.85 (2.60-13.15) | <0.001 |
| Anti-coagulants drug |  |  |
| No | Ref |  |
| Yes | 3.54 (1.28-9.79) | 0.016 |
| Adrenal cortical steroids |  |  |
| No | Ref |  |
| Yes | 0.91 (0.23-3.55) | 0.889 |
| Diabetes drug |  |  |
| No | Ref |  |
| Yes | 1.82 (1.06-3.13) | 0.030 |

OR: odds ratio; CI: confidence interval; Ref: reference; CVD: Cardiovascular disease; CKD: chronic kidney disease; GED: general equivalent diploma; AA: Associate of Arts
